# Secretory carcinoma of minor salivary glands of buccal mucosa: A case report and review of the literature

**DOI:** 10.1016/j.ijscr.2023.108357

**Published:** 2023-05-26

**Authors:** Noshad Ali Langah, Abdul Ahad, Shayan Khalid Ghaloo, Muhammad Faisal, Raza Tasawar Hussain, Fareed Akbar Shah

**Affiliations:** a Shaukat Khanum Memorial Cancer Hospital, Lahore, Pakistan; b Taibah University Madinah Al Munawwarah, Saudi Arabia

**Keywords:** Minor salivary gland tumor, Neck dissection, Buccal mucosa

## Abstract

**Introduction and importance:**

Secretory carcinoma (SC) is an uncommon salivary gland neoplasm of the oral cavity that microscopically may mimic acinic cell carcinoma (ACC) and mucoepidermoid carcinoma (MEC). Secretory carcinoma (SC) of the salivary gland has been recently added in fourth edition of the head and neck world health organization. Most of these tumors are located on the parotid gland with very few cases reported in the minor salivary glands of the buccal mucosa.

This work has been reported in line with the SCARE criteria.

**Presentation of case:**

A 42 years old hypertensive male, shop keeper by occupation, with no prior addiction history, no dental extraction or trauma, presented with complaint of nodular lesion on left buccal mucosa for five years. On Clinical examination, adequate mouth opening, dentulous patient with 2.4 × 2 cm well circumscribed, nodular, non-tender, benign looking lesion was observed on left buccal mucosa near upper alveolus. Overlying mucosa appeared normal with no clinically palpable cervical lymphadenopathy. Histopathology revealed salivary gland neoplasm favoring secretory carcinoma. MRI scan showed lobulated enhancing nodular lesion arising from left buccal mucosa of size 2.3 ∗ 1.3 ∗ 1.7 cm, close to left superior alveolus without involving any cortical areas of marrow infiltration, with bilateral symmetrical level IIa reactive cervical nodes. Wide local excision and ipsilateral selective neck dissection [level 1, 2, 3] was done. Post-operative period was smooth with no complain of paresthesia observed. The final histopathology report showed secretory carcinoma. Two out of six lymph nodes from level I were positive for metastatic carcinoma with no extra nodal extension. Final stage of the tumor was pT1N2bMx.

Patient underwent post-operative adjuvant radiotherapy for period of 6 weeks, received total 30 fractions and total dose of 6000 centigray.

**Clinical discussion:**

SC behaved clinically an indolent being painless and having long duration of symptoms with normal overlying mucosa. But histopathologically there was cervical node metastasis. That changed final staging and added adjuvant treatment for this patient. The discrepancy in clinical and pathological diagnosis might be due to the indolent clinical behavior of SC arising in the minor salivary gland of buccal mucosa. In the present case, the absence of zymogen granules and presence of microcytic pattern with eosinophilic cytoplasm and eosinophilic secretory material were suggestive of SC.

**Conclusion:**

This case report represents a rare case of SC of minor salivary glands of buccal mucosa, which was indolent as per clinical presentation but on final histopathological report it had cervical nodal metastasis that changed the final stage of the disease, for which adjuvant radiotherapy was needed. Although Secretory carcinomas are generally considered having a favorable prognosis and are regarded as low- grade carcinomas with limited number of recurrence and cervical nodal metastasis, but sometimes they do metastasize to cervical nodes for which accurate and timely intervention in the form of neck dissection may be performed to establish final staging and start additional treatment modality if required for better outcome of the disease.

## Highlights

• SC is a tumor of major salivary glands that is not very frequent in minor salivary glands of buccal mucosa.
• Lymph node metastasis in the neck is rarely seen in secretory carcinoma.
• Clinical and radiological correlation with tissue diagnosis is important.

## 1 Introduction and importance

Secretory carcinoma [SC] also known as mammary analogue secretory carcinoma, initially described by Skalova A et al. in 2010 [1]. SC has been recently included in fourth edition of the Head and Neck World Health organization blue book 2017 [1]. Most of the cases of this carcinoma have been located in major salivary parotid gland, and only some are reported in minor salivary glands [[2], [3], [4]]. Herein, we report a case of SC in the minor salivary gland of the buccal mucosa and present a brief review of literature regarding this condition.

## 2 Presentation of case

**Fig. 1:**
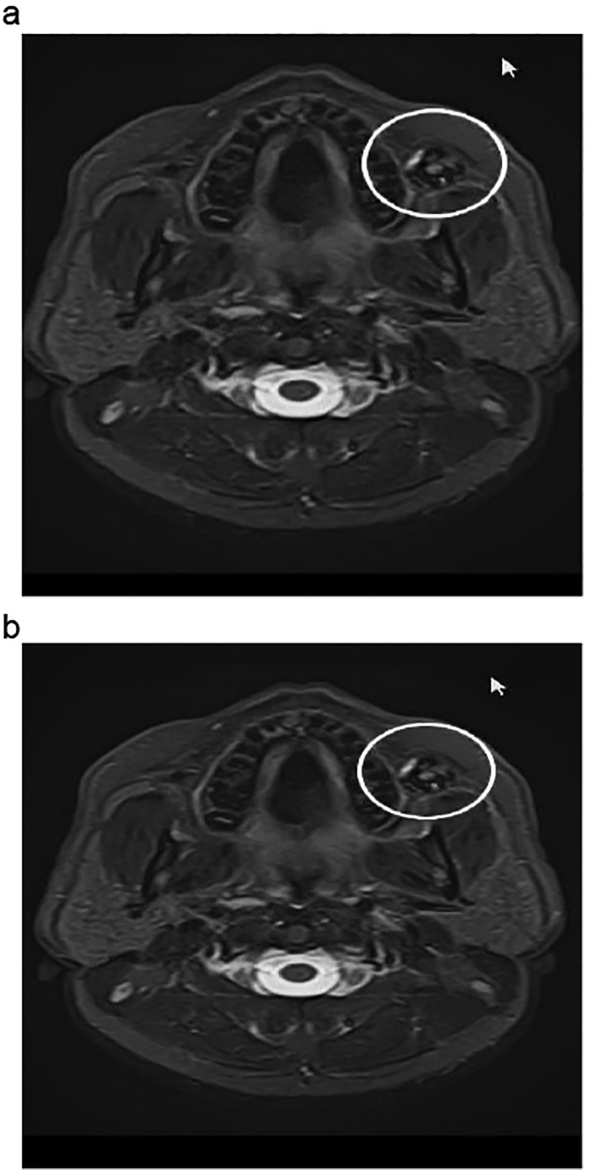
a: MRI of the patient showing enhancing nodular lesion arising from left buccal mucosa. b: MRI of the patient showing enhancing nodular lesion arising from left buccal mucosa.

**Fig. 2:**
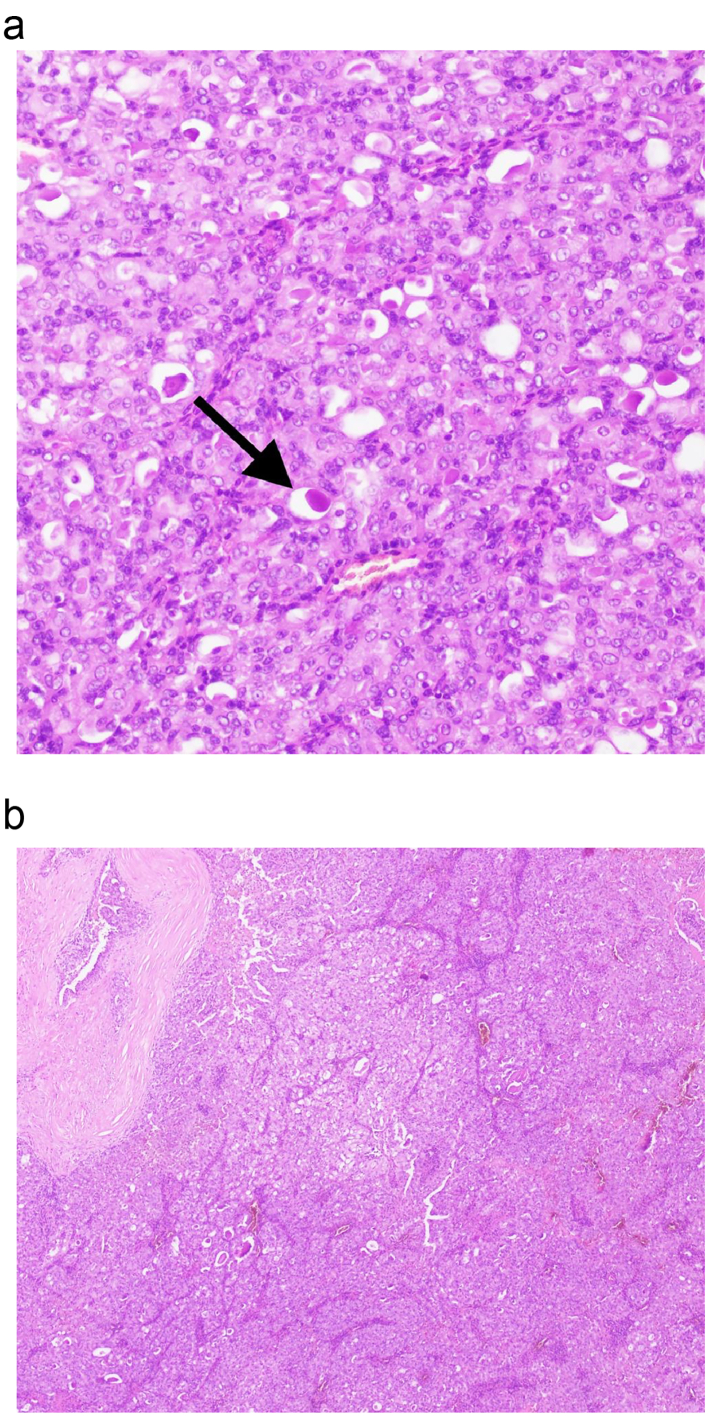
a: Showing monomorphic, round, vesicular nuclei and small discohesive nucleoli and pale eosinophilic colloid like secretions present intraluminal. b: Neoplasm composed of eosinophilic tumor cells arranged in papillary architecture.

A 42 years old hypertensive male, shop keeper by occupation, with no prior addiction history, no dental extraction or trauma, presented with complaint of nodular lesion on left buccal mucosa for five years. On Clinical examination, adequate mouth opening, dentulous patient having 2.4 × 2 cm well circumscribed, nodular, non-tender, benign looking lesion on left buccal mucosa near upper alveolus. Overlying mucosa appeared normal with no clinically palpable cervical lymphadenopathy. Patient was referred from outside hospital to oncology clinic with histopathology report already done from outside, which revealed salivary gland neoplasm favoring secretory carcinoma. Review of those slides were done from histopathology department of hospital. The report revealed a neoplasm composed of eosinophilic tumor cells arranged in papillary architecture. Immunohistochemistry showed positive stain for GATA3, MUC4, and negative for AR. Final diagnosis was secretory carcinoma of salivary gland of buccal mucosa. Contrast magnetic resonance imaging scan (Fig. 1a, b) showed lobulated enhancing nodular lesion arising from left buccal mucosa of size 2.3 ∗ 1.3 ∗ 1.7 cm, close to left superior alveolus without involving any cortical areas of marrow infiltration, with bilateral symmetrical level IIa reactive cervical nodes. Multidisciplinary team meeting of hospital advised for primary resection and ipsilateral neck dissection. Wide local excision and ipsilateral selective neck dissection level 1, 2, 3, and dental extraction of carious teeth was done. Intra operative findings included submucosal encapsulated tumor abutting the buccal fat, and enlarged nodes at level I and II. Frozen section for deep margin of lesion, came out to be negative for malignancy. Surgery and post-operative recovery periods were uneventful. The final histopathology report showed secretory carcinoma, tumor size of 12 mm, all margins free of tumor, no lympho vascular or *peri* neural invasion was seen. The histopathology slide (Fig. 2a, b) showed monomorphic, round, vesicular nuclei and small discohesive nucleoli and pale eosinophilic colloid like secretions present intraluminal. Two out of six lymph nodes from level I were positive for metastatic carcinoma with no extra nodal extension. Level II and III were negative for metastasis. Final stage was pT1N2bMx.

Based on histopathology report patient underwent post-operative radiotherapy for period of 6 weeks received 30 fractions and total dose of 6000 centigray.

Post treatment patient had no loco-regional recurrence for six months and was kept on regular follow up.

## 3 Discussion

Intraoral salivary gland malignancy make up 6 % of all head and neck malignancies [5]. One such rare and distinct variant was first described by Skalova et al. [2] as mammary analogue secretory carcinoma, recently renamed secretory carcinoma (SC) of salivary glands [3]. Recent reviews estimates that SC forms less than 0.3 % of all salivary gland malignancies [6]. Fewer than 300 cases of SC have been reported in the literature with 70 % of these cases involving the major salivary glands, primarily the parotid gland with less frequency in minor salivary glands [[7], [8], [9]]. SC arising in intraoral salivary locations like palate and labial mucosa have rarely been reported [6]. One of the literature review showed that 63 cases of SC of minor salivary glands have been reported. Among them, only 15 cases were found in buccal mucosa. The lip was the most affected site (21 cases) followed by palate (17 cases). Two cases were reported in tongue, labial mucosa, and retro molar gingiva each and 1 case in floor of mouth. Most of the tumors presented as a slow growing and painless mass. The only aggressive tumor was in hard palate [10]. Lymph node metastases occurred in only 4 patients [2,[10], [11], [12]] and local recurrence was reported in 4 patients [10,11,13,and]. These clinical features indicate that SC in the minor salivary glands may have a good prognosis with rare recurrence and lymph node metastases. SC in the head and neck region develops in individuals in their 40 s, however, a childhood-onset case has similarly been reported in literature [2]. SC has no sex predilection. The true frequency of occurrence and risk factors are unclear in literature because SC is a recently described disease entity.SC in this case behaved clinically an indolent being painless and having long duration of symptoms with normal overlying mucosa. But histopathologically there was cervical node metastasis. This discrepancy in clinical and pathological diagnosis might be due to the indolent clinical behavior of SC arising in the minor salivary gland of buccal mucosa. The differential diagnosis of SC includes AcCC, low-grade mucoepidermoid carcinoma, and polymorphous low-grade adenocarcinoma [3]. Most of the cases of SC were previously diagnosed as AcCC because of their histopathological similarities. Nevertheless, some histomorphological findings are more common in SC than in AcCC. Few authors reported that the presence of papillary cystic and microcytic patterns with vacuolated cells is characteristic of SC [14,15]. Hemosiderin deposition was also more commonly observed in SC than in AcCC [16]. In the present case, the absence of zymogen granules and presence of microcytic pattern with eosinophilic cytoplasm and eosinophilic secretory material were suggestive of SC rather than AcCC.

ACCs demonstrate positive immunoreactivity towards DOG1. Approximately 90 % of ACCs exhibit diffuse positivity towards DOG 1 making it a very reliable marker [17]. The oncocytic variant of MECs also shares similar histological features with SC. Most MEC exhibit squamous differentiation with abundant mucous cells in the background. These epidermoid cells usually express CK5, CK6, CK7, CK8, CK14, CK18, CK19 [18].

ETV6-NTRK3 fusion protein can be detected by cytogenetic analysis through FISH or RT-PCR in Secretory Carcinoma. Although, ETV6-NTRK3 translocation is the most common cytogenetic alteration among SC (80 %), 15–20 % of cases may exhibit rearrangements between ETV6 and RET gene [19]. The histological, immunohistochemical, and genetic appearance of SC of salivary gland is similar to that of breast secretory carcinoma. This work has been reported in line with the SCARE criteria [20].

## 4 Conclusion

This case report represents a rare case of SC of minor salivary glands of buccal mucosa, which was indolent as per clinical presentation but on final histopathological report it had cervical nodal metastasis that changed the final stage of the disease, for which adjuvant radiotherapy was needed. Although Secretory carcinomas are generally considered having a favorable prognosis and are regarded as low-grade carcinomas with limited number of recurrence and cervical nodal metastasis, but sometimes they do metastasize to cervical nodes for which accurate and timely intervention in the form of neck dissection may be performed to establish final staging and start additional treatment modality if required for better outcome of the disease.

## Ethical approval

Ethical Approval was waived by the author's institution.

## Sources of funding

Not applicable.

## CRediT authorship contribution statement

Dr. Noshad Ali Langah – Study concept, writing the paper.

Dr. Abdul Ahad – Writing the paper, Data collection.

Dr. Shayan Khalid Ghaloo – Writing the paper, Data collection.

Dr. Muhammad Faisal – Writing the paper.

Dr. Raza Tasawar Hussain – Writing the paper.

Dr. Fareed Akbar shah- Writing the paper.

## Patient consent

Written informed consent was obtained from the patient for publication of this case report and accompanying images. A copy of the written consent is available for review by the Editor-in-Chief of this journal on request.

## Guarantor

Dr. Raza Tasawar Hussain.

## Research registration

Not applicable.

## Declaration of competing interest

Not applicable.
